# Elucidating the causal relationship between gut microbiota, metabolites, and diabetic nephropathy in European patients: Revelations from genome-wide bidirectional mendelian randomization analysis

**DOI:** 10.3389/fendo.2024.1391891

**Published:** 2025-01-08

**Authors:** Siyuan Song, Li Ning, Jiangyi Yu

**Affiliations:** ^1^ Department of Endocrinology, Jiangsu Province Hospital of Chinese Medicine, Affiliated Hospital of Nanjing University of Chinese Medicine, Nanjing, China; ^2^ Department of Gynecology, Jiangsu Provincial Hospital of Traditional Chinese Medicine, Affiliated Hospital of Nanjing University of Chinese Medicine, Nanjing, China

**Keywords:** mendelian randomization analysis, gut microbiota, metabolites, diabetic nephropathy, bidirectional

## Abstract

**Objective:**

Previous observational studies suggest a potential link between gut microbiota, metabolites, and diabetic nephropathy. However, the exact causal relationship among these factors remains unclear.

**Method:**

We conducted a two-sample bidirectional Mendelian randomization study using summary statistics from the IEU OpenGWAS Project database to investigate gut microbiota, metabolites, and diabetic nephropathy. A range of methods, including inverse variance weighting, MR-Egger, weighted median, and simple median, were applied to examine causal associations. Sensitivity analyses were performed to assess the robustness of the results. Additionally, reverse Mendelian randomization analysis was conducted, treating significant gut microbiota as the outcome, to evaluate effects and perform sensitivity testing. This comprehensive approach provided an in-depth assessment of the interactions among gut microbiota, metabolites, and diabetic nephropathy.

**Result:**

The Inverse Variance Weighted estimates revealed that the abundance of *Lachnospiraceae, Parasutterella*, and *Eubacterium* exhibited negative causal effects on diabetic nephropathy, while *Coprococcus, Sutterella, Faecalibacterium prausnitzii*, and *Bacteroides vulgatus* showed protective causal effects against the condition. However, reverse Mendelian randomization analysis did not identify any significant associations between diabetic nephropathy and the identified gut microbiota. Furthermore, the estimates indicated that Cholesterol, Pyridoxate, Hexanoylcarnitine, X-12007, Octanoylcarnitine, 10-nonadecenoate (19:1n9), X-12734, and the average number of double bonds in a fatty acid chain had negative causal effects on diabetic nephropathy. In contrast, Methionine, Glycodeoxycholate, X-06351, 1-stearoylglycerol (1-monostearin), 5-dodecenoate (12:1n7), X-13859, 2-hydroxyglutarate, Glycoproteins, Phospholipids in IDL, and the concentration of small HDL particles demonstrated protective causal effects. Notably, sensitivity analyses did not detect any heterogeneity or horizontal pleiotropy, ensuring the robustness of the findings.

**Conclusion:**

Modulating gut microbiota diversity and composition offers a promising strategy for improving the incidence and prognosis of diabetic nephropathy. This highlights the need for future clinical trials focusing on microbiome-based interventions, potentially utilizing microbiome-dependent metabolites. Such approaches could transform the treatment and management of diabetic nephropathy and its associated risk factors, paving the way for more effective therapeutic strategies to combat this debilitating condition.

## 1 Introduction

Diabetic nephropathy (DN) is a common complication of type 2 diabetes mellitus (T2DM). It is widely recognized that interactions among inflammatory cytokines, endocrine factors, immune responses, oxidative stress, and abnormal lipid metabolism play a critical role in disrupting cellular structure, promoting insulin resistance, and impairing microvascular function (1). Elevated glucose levels, inflammation, and vascular damage are pivotal drivers of pathological alterations in the kidneys, emerging as primary factors in the progression of DN (2). Notable pathological manifestations encompass glomerular hypertrophy, mesangial expansion, and podocyte injury, which, with the gradual decline of renal function, culminate in glomerular sclerosis and interstitial fibrosis (3). As the glomerular filtration rate diminishes, various toxins accumulate in the bloodstream, with the intestine serving as the principal route for excretion (4). As a result, toxins disrupt the intestinal pH balance and weaken the innate intestinal barrier, allowing microorganisms and their byproducts to enter the bloodstream. This process triggers systemic inflammatory responses, further accelerating the decline in renal function (5). Studies by Li (6) underscored the pivotal role of gut microbiota (GM) in modulating renal function in DN mouse models. Additionally, research conducted by LUN (7) revealed diminished probiotics and heightened pathogenic bacteria in the intestines of DN patients, coupled with elevated inflammatory factors in systemic circulation, which expedited kidney injury-induced inflammation. Moreover, Tao (8) observed a significant increase in Escherichia coli-Shigella abundance at the genus level in DN patients, exacerbating intestinal permeability by breaching the intestinal epithelial barrier (9). Consequently, the production of ethanol further disrupted fatty acid metabolism upon entry into the liver via the bloodstream (10).

The metabolites originating from the GM are widely acknowledged as pivotal regulators of various bodily functions and metabolic processes. Imbalances in GM are characterized by shifts and disparities in the composition of the metabolite community, which have been closely associated with DN (11). GM exerts its influence on the host’s immune and physiological functions primarily through the production of metabolites (12). Notably, studies highlight the importance of metabolites such as short-chain fatty acids (SCFAs), bile acids, and tryptophan metabolites as key mediators of the GM influence on the host. Once these metabolites cross the intestinal barrier and enter systemic circulation, they can contribute to the pathogenesis of DN by regulating inflammation, oxidative stress, and fibrosis (13). For instance, the promotion of *Plasmodium* and *Bifidobacterium* enrichment, which are producers of SCFAs, and subsequent elevation of SCFA levels have been linked to reduced expression of genes encoding inflammatory cytokines, chemokines, and fibrogenic proteins in DN (14). However, due to potential confounders and reverse causality inherent in observational studies, the causal relationship between gut microbiota, metabolites, and the risk of diabetic nephropathy remains unclear.

**Figure 1:**
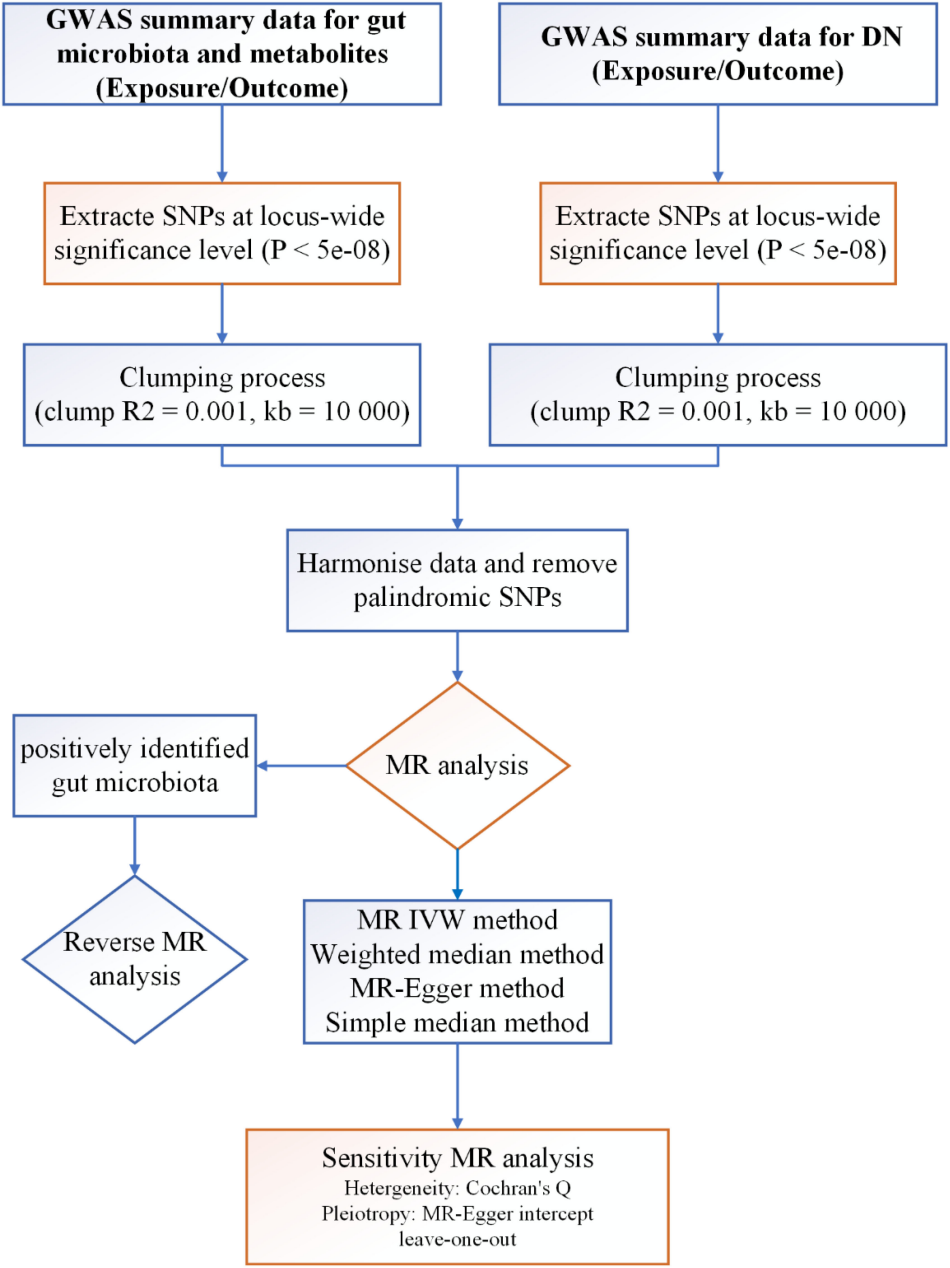
Protocol of study procedure.

The core principle of Mendelian randomization (MR) research lies in utilizing genetic variants, particularly single nucleotide polymorphisms (SNPs), as instrumental variables to investigate exposures. This approach enables researchers to evaluate the causal relationship between an exposure and an outcome. MR provides a unique advantage in clarifying the causal link between phenotypes and diseases by relying on the Mendelian randomization principle, which asserts that alleles are randomly inherited from parents to offspring. This random distribution minimizes the impact of common confounders, such as environmental factors, socio-economic status, and behavioral influences (15). In our study, we harnessed genome-wide association study (GWAS) data and conducted two-sample MR analysis to investigate the causal association between GM, metabolites, and the risk of DN, thereby furnishing genetic substantiation for their interrelation. The schematic representation of our study protocol is delineated in **Figure 1**.

## 2 Materials and method

### 2.1 Study design

**Figure 2:**
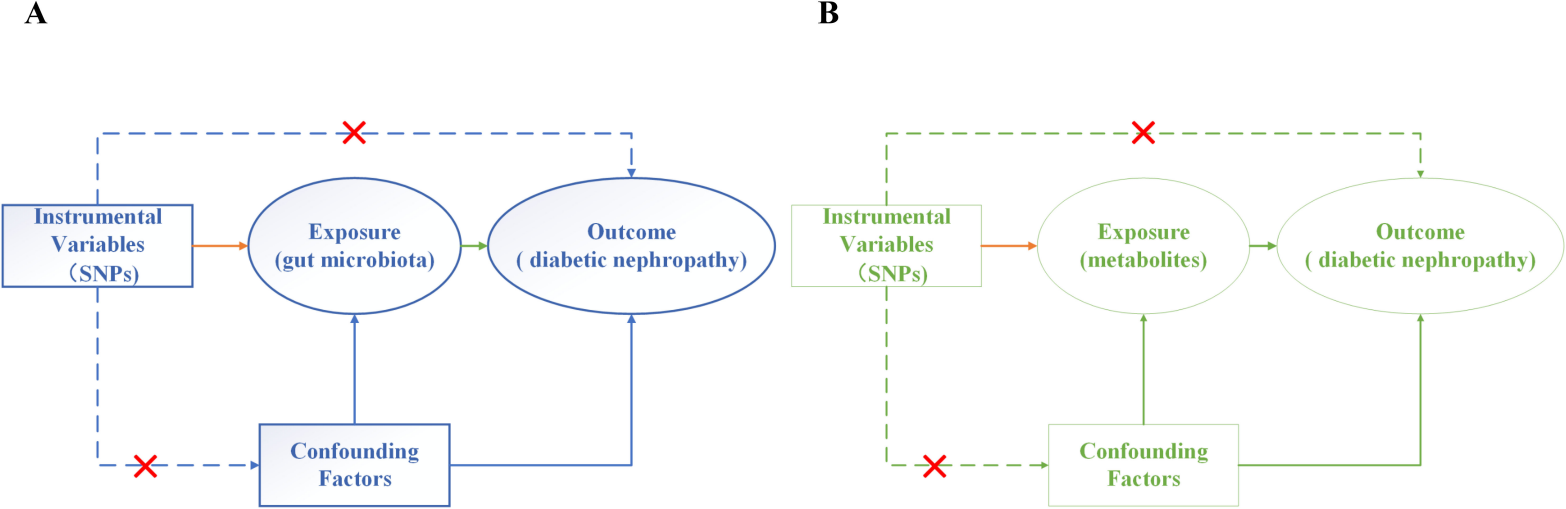
Three assumptions of MR analysis. **(A)** Gut microbiota and diabetic nephropathy; **(B)** Metabolites and diabetic nephropathy.

In this paper, we devised a two-sample bidirectional MR analysis to scrutinize the causal relationship between GM, metabolites, and DN. Leveraging GWAS data sourced for GM, metabolites, and DN, the effectiveness of instrumental variables (IVs) hinges on satisfying three critical assumptions (**Figures 2A, B**): (1) Relevance Hypothesis: This foundational premise asserts that the chosen independent Instrumental Variables (IVs) maintain a direct and substantive linkage to the exposure factors under scrutiny. (2) Independence Hypothesis: This tenet mandates that the selected IVs remain devoid of any association with confounding variables mediating the relationship between the exposure and outcome. To diligently uphold this assumption, we judiciously leveraged the PhenoScanner (http://www.phenoscanner.medschl.cam.ac.uk/) database to meticulously identify all eligible SNPs. Subsequent to this, we meticulously eliminated any confounding factors correlated with DN and DN-related SNPs. (3) Exclusion Restriction Hypothesis: In accordance with this postulate, the chosen IVs should not wield any influence on the analytical outcomes unless they are intrinsically associated with the exposure variable (16).

In our investigation, we meticulously employed a stringent significance threshold for SNPs across the entire genome (*P* < 5e-08) and implemented measures for linkage disequilibrium (R2 = 0.001, kb = 10 000) to ascertain potential IVs. These meticulously curated IVs were selected to guarantee both their independence and relevance to the study’s objectives (17).

### 2.2 Exposure data

A thorough search was conducted within the IEU OpenGWAS Project (gwas.mrcieu.ac.uk) using “gut microbiota” and “metabolites” as key terms to obtain relevant data. This extensive search led to the identification of 418 genetic loci associated with gut microbiota and 575 genetic loci linked to metabolites. All identified loci met stringent significance thresholds (*P* < 5e-08, R² = 0.001, kb = 10,000). These carefully selected genetic loci were then used as IVs to represent the exposure variables of gut microbiota and metabolites in our study.

In order to gauge the robustness of correlation between the identified genetic loci and their corresponding exposure factors, we meticulously computed the F value for each SNP using the prescribed formula F = β^2/SE^2, where β signifies the allele effect value and SE denotes the standard error. A threshold of F value surpassing 10 serves as a well-established benchmark denoting unbiased IVs (18). This rigorous criterion underscores the accuracy and effectiveness of our IV selection method. To further ensure the integrity of our analysis, palindrome SNPs were systematically identified and removed using palindrome sequence detection algorithms. This precaution was taken to mitigate the potential influence of alleles on our results, thereby enhancing the reliability and robustness of our findings.

### 2.3 Outcome data

The outcome data used in this study, identified by the unique identifier ebi-a-GCST90018832, were carefully sourced from the IEU OpenGWAS Project website (gwas.mrcieu.ac.uk). This dataset, specifically focused on DN, includes a substantial sample size of 452,280 individuals and a comprehensive catalog of 24,190,738 SNPs.

As the study relies solely on publicly available data from reputable repositories, no additional ethical approval or consent was required under the ethical framework guiding this research.

### 2.4 Statistical analysis

#### 2.4.1 MR analysis

In our endeavor to elucidate the intricate relationship between GM, metabolites, and DN, we employed a diverse array of well-established MR methodologies, including: Inverse Variance Weighted (IVW): Revered as the cornerstone of MR analysis, IVW method boasts robust detection capabilities (19). Nonetheless, it may inadvertently overlook pleiotropic effects at the genetic level (20). MR-Egger: Diverging from IVW, MR-Egger exhibits a diminished sensitivity to the validity of IVs and showcases prowess in detecting horizontal pleiotropy through its intercept (21). Weighted Median (WME): This approach mandates that a minimum of 50% of the weights originate from valid IVs to bolster the precision of IVW estimates. Simple Median (SME): SME delineates SNPs with analogous values into clusters, leveraging the cluster harboring the greatest number of SNPs to assess causality (22).

To enhance the robustness and reliability of our findings, we complemented these methodologies with forest plots for visual representation. In the absence of horizontal pleiotropy, the integrity of the IVW method remains intact, providing robust analytical outcomes compared to alternative approaches. Statistical significance was determined at the conventional threshold of *P* < 0.05, indicating a causal relationship between the exposure variables (gut microbiota, metabolites) and the outcome variable (diabetic nephropathy) (23). This meticulously curated MR analysis approach empowered us to rigorously scrutinize the potential causal interlinks between GM, metabolites, and DN, thereby enriching our comprehension of their intricate interplay within the realm of DN.

#### 2.4.2 Sensitivity analysis

To strengthen the robustness and credibility of our findings, we conducted a comprehensive sensitivity analysis using a range of well-established methodologies. The MR-Egger Intercept Method was applied to detect horizontal pleiotropy, providing valuable insights into potential biases in the causal estimates (24). Leave-One-Out (LOO) Analysis: This meticulous approach was employed to scrutinize the sensitivity of our findings by systematically evaluating the impact of individual SNP on the inferred causal relationship (25). Cochran’s Q Statistics: Heterogeneity was carefully assessed using Cochran’s Q statistics, with a significance threshold of *P* < 0.05 indicating the presence of heterogeneity. This prompted further investigation using the IVW method with a random-effects model to evaluate consistency. Funnel Plot: This graphical tool was used for a visual assessment of heterogeneity. A symmetrical distribution of SNPs across the plot suggested no significant heterogeneity in the results (26).

#### 2.4.3 Statistical software

The entirety of MR analyses was meticulously performed utilizing R (version 4.3.1) alongside the TwoSampleMR package, thereby upholding stringent statistical protocols and fostering a nuanced comprehension of the yielded outcomes.

## 3 Results

### 3.1 Selection of IVs

**Figure 3:**
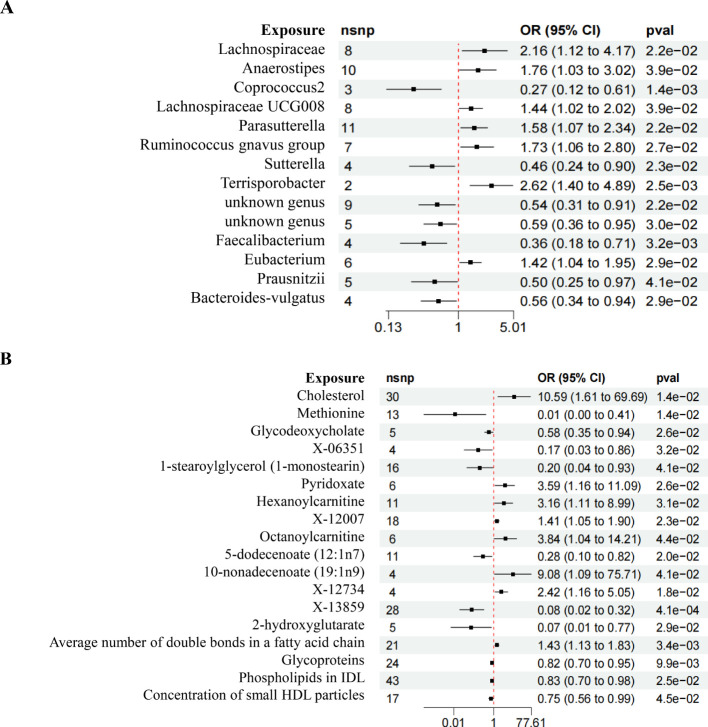
Forest plot of MR results. **(A)** Forest plot depicting the MR results of GM on DN. **(B)** Forest plot illustrating the MR results of Metabolites on DN.

Following rigorous screening criteria for IVs, a total of 8, 10, 3, 8, 11, 7, 4, 2, 9, 5, 4, 6, 5, and 4 SNPs were extracted from the *Lachnospiraceae, Anaerostipes, Coprococcus2, Lachnospiraceae UCG008, Parasutterella, Ruminococcus gnavus group, Sutterella, Terrisporobacter, unknown genus (id.2071), unknown genus (id.2755), Faecalibacterium, Eubacterium, Prausnitzii, and Bacteroides-vulgatus* as IVs (**Figure 3A**). Similarly, from the metabolites including Cholesterol, Methionine, Glycodeoxycholate, X-06351, 1-stearoylglycerol (1-monostearin), Pyridoxate, Hexanoylcarnitine, X-12007, Octanoylcarnitine, 5-dodecenoate (12:1n7), 10-nonadecenoate (19:1n9), X-12734, X-13859, 2-hydroxyglutarate, the average number of double bonds in a fatty acid chain, Glycoproteins, Phospholipids in IDL, and the concentration of small HDL particles, a total of 30, 13, 5, 4, 16, 6, 11, 18, 6, 11, 4, 4, 28, 5, 21, 24, 43, and 17 SNPs were identified as IVs (**Figure 3B**). All IVs in this study had F statistics greater than 10, indicating minimal bias from weak instruments and ensuring the robustness of our analyses (**Supplementary Table 1**).

### 3.2 MR analysis

**Figure 4:**
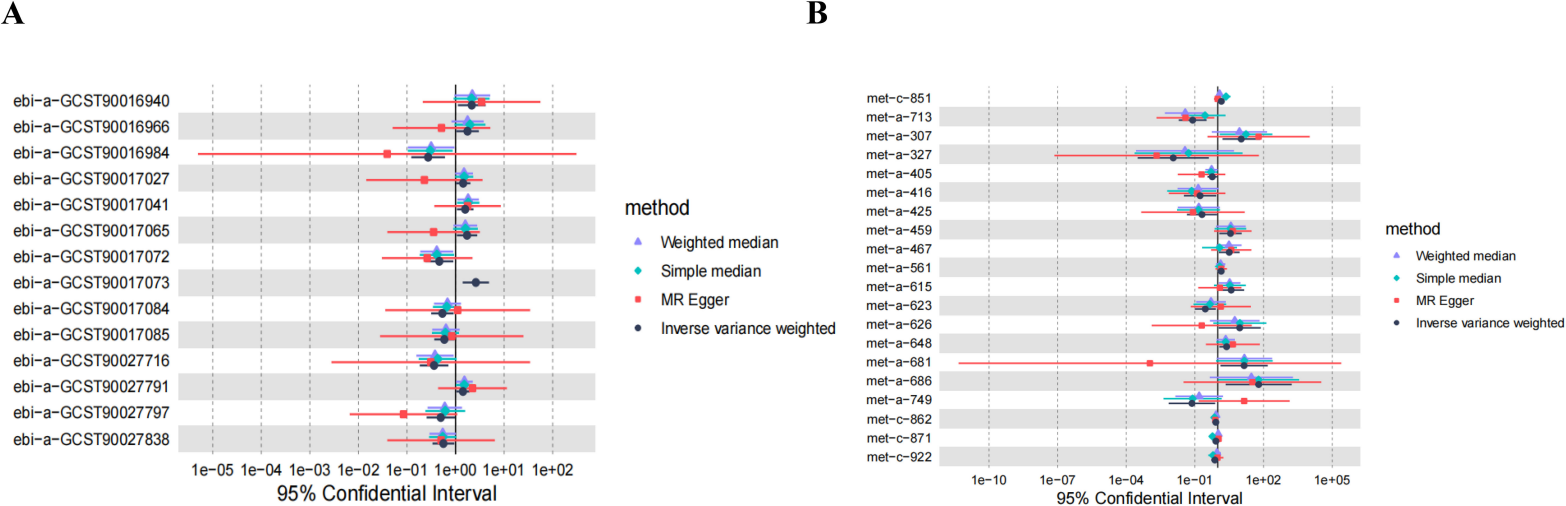
MR estimates of causal effects. **(A)** Forest plot displaying the estimates of causal effects of GM on DN. **(B)** Forest plot presenting the estimates of causal effects of Metabolites on DN.

The IVW analysis revealed compelling insights, showing that certain microbial taxa have a significant causal relationship with the risk of DN. Specifically, *Lachnospiraceae* (*OR* = 2.156, *95%CI* 1.115-4.169, *P* = 0.022), *Parasutterella* (*OR* = 1.435, *95%CI* 1.018-2.024, *P* = 0.039), and *Eubacterium* (*OR* = 1.421, *95%CI* 1.037-1.949, *P* = 0.028) were associated with an increased risk of DN. Conversely, *Coprococcus2* (*OR* = 1.10, *95%CI* 1.01-1.19, *P* = 2.5e-02), *Sutterella* (*OR* = 1.725, *95%CI* 1.064-2.796, *P* = 0.026), *unknown genus (id.2755)* (*OR* = 0.588, *95%CI* 0.365-0.949, *P* = 0.029), *Faecalibacterium* (*OR* = 0.361, *95%CI* 0.183-0.712, *P* = 0.003), *Prausnitzii* (*OR* = 0.496, *95%CI* 0.253-0.973, *P* = 0.041), and *Bacteroides-vulgatus* (*OR* = 0.592, *95%CI* 0.335-0.943, *P* = 0.029) were associated with a reduced risk of DN (**Figure 4A**).

The comprehensive analysis using the IVW method revealed insightful causal associations between metabolites and the risk of DN. Remarkably, the Average number of double bonds in a fatty acid chain (*OR* = 1.434, *95%CI* 1.126-1.825, *P* = 0.003), Cholesterol (*OR* = 10.591, *95%CI* 1.609-69.692, *P* = 0.014), Pyridoxate (*OR* = 3.588, *95%CI* 1.161-11.087, *P* = 0.026), Hexanoylcarnitine (*OR* = 3.164, *95%CI* 1.114-8.988, *P* = 0.030), X-12007 (*OR* = 1.410, *95%CI* 1.407-1.898, *P* = 0.023), Octanoylcarnitine (*OR* = 3.843, *95%CI* 1.039-14.306, *P* = 0.043), and X-12734 (*OR* = 2.423, *95%CI* 1.163-5.047, *P* = 0.018) were found to be causally linked to an increased risk of DN. Conversely, X-13859 (*OR* = 0.079, *95%CI* 0.019-0.324, *P* = 0.000), Methionine (*OR* = 0.011, *95%CI* 0.000-0.407, *P* = 0.014), Glycodeoxycholate (*OR* = 0.575, *95%CI* 0.354-0.936, *P* = 0.025), X-06351 (*OR* = 0.168, *95%CI* 0.033-0.855, *P* = 0.031), 1-stearoylglycerol (1-monostearin) (*OR* = 0.204, *95%CI* 0.044-0.933, *P* = 0.040), Glycoproteins (*OR* = 0.819, *95%CI* 0.704-0.953, *P* = 0.009), and Concentration of small HDL particles (*OR* = 0.745, *95%CI* 0.559-0.993, *P* = 0.045) were associated with a reduced risk of DN (**Figure 4B**).

**Figure 5:**
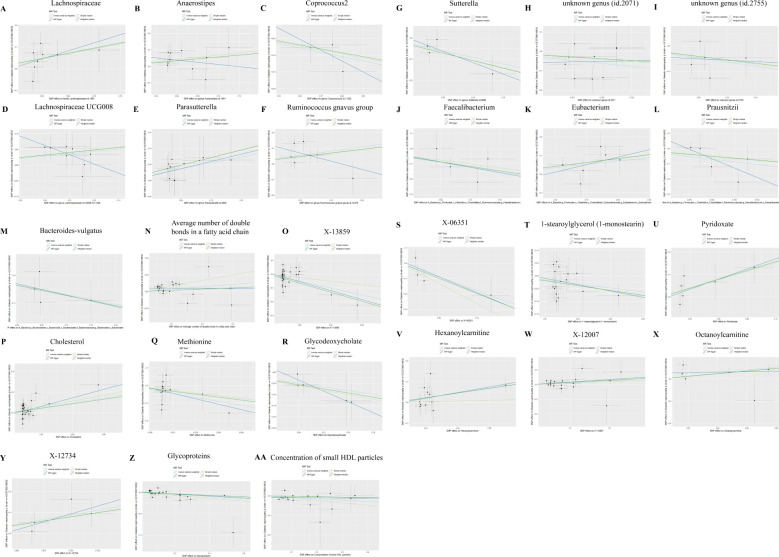
Scatter plots of SNP analysis. The X-axis represents the influence of SNPs on GM or Metabolites, while the Y-axis represents the influence of SNPs on DN. Each black dot represents a single SNP, with the line segment indicating the 95%CI. The slope of the straight line represents the causal estimation of the MR method. Specifically, the light blue line represents the Inverse Variance Weighted (IVW) method, the blue line represents MR-Egger, the green line represents the Weighted Median (WME) method, and the light line represents the Simple Median (SME) method. **(A)** Lachnospiraceae; **(B)** Anaerostipes; **(C)** Coprococcus2; **(D)** Lachnospiraceae UCG008; **(E)** Parasutterella; **(F)** Ruminococcus gnavus group; **(G)** Sutterella; **(H)** unknown genus (id.2071); **(I)** unknown genus (id.2755); **(J)** Faecalibacterium; **(K)** Eubacterium; **(L)** Prausnitzii; **(M)** Bacteroidesvulgatus; **(N)** Average number of double bonds in a fatty acid chain; **(O)** X-13859; **(P)** Cholesterol; **(Q)** Methionine; **(R)** Glycodeoxycholate; **(S)** X-06351; **(T)** 1-stearoylglycerol (1-monostearin); **(U)** Pyridoxate; **(V)** Hexanoylcarnitine; **(W)** X-12007; **(X)** Octanoylcarnitine; **(Y)** X-12734; **(Z)** Glycoproteins: **(AA)** Concentration of small HDL particles.

In the analysis involving *Anaerostipes, Lachnospiraceae UCG008, Ruminococcus gnavus group, unknown genus (id.2071)*, 5-dodecenoate (12:1n7), X-13477, 10-nonadecenoate (19:1n9), Oleoylcarnitine, Phospholipids in IDL, 2-hydroxyglutarate, and DN, discrepancies emerged between the total effect values of MR-Egger and IVW, warranting their exclusion from further consideration. The divergent outcomes observed between these two analytical methods underscored the need for cautious interpretation. Subsequent analyses employing the WME and SME approaches affirmed the necessity of their exclusion, thus consolidating the robustness of our conclusions. Although the results derived from MR-Egger regression analysis did not attain statistical significance, their directional alignment with the preceding analyses lent additional support to the overarching findings (**Figures 5A–AA**). Given the reliability and consistency of the IVW analysis, supported by the forest plot, it is reasonable to conclude that GM and metabolites are indeed associated with the onset of DN.

### 3.3 Sensitivity analysis

Cochran’s Q test showed no evidence of heterogeneity among the included instrumental variables (*P* > 0.05). Additionally, the intercept test of MR-Egger regression indicated that pleiotropy was unlikely to bias the results (*P* > 0.05) (**Supplementary Table 2**). Funnel plots demonstrated minimal susceptibility to potential confounding factors, further reinforcing the robustness of the causality assessments (**Supplementary Figures 1A–W**). The LOO sensitivity analysis confirmed the stability of the findings, as excluding individual SNPs did not significantly affect the results (**Supplementary Figures 2A–W**).

### 3.4 Bidirectional MR analysis

**Figure 6:**
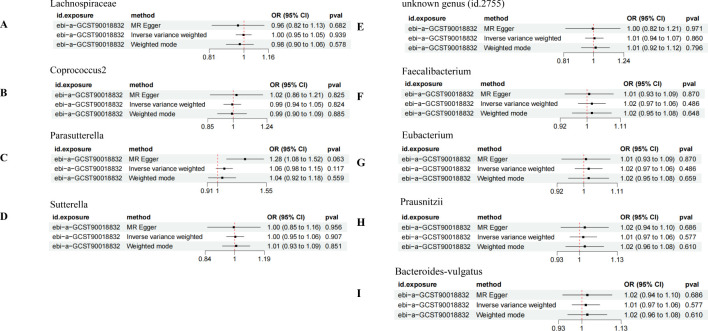
Bidirectional MR analysis of DN on the positively identified GM. **(A)** Lachnospiraceae; **(B)** Coprococcus2; **(C)** Parasutterella; **(D)** Sutterella; **(E)** unknown genus (id.2755); **(F)** Faecalibacterium; **(G)** Eubacterium; **(H)** Prausnitzii; **(I)** Bacteroides-vulgatus.

In the bidirectional MR analysis, the results of the reverse MR analysis showed no causal relationship between DN and the increased risk associated with the positively identified GM, suggesting that DN does not influence the abundance of these GM species (**Figures 6A–I**).

### 3.5 FDR correction

To minimize the likelihood of false positives, we implemented false discovery rate (FDR) correction to adjust the P-values. Following correction, our analysis unveiled that only Faecalibacterium displayed a significant negative correlation with DN risk (*q value* = 0.05), while the Average number of double bonds in a fatty acid chain showed a positive correlation with DN risk (*q value* = 0.08). However, the remaining GM species and metabolites still showed potential associations with DN. These findings offer novel insights into potential therapeutic approaches and deepen our understanding of the mechanisms underlying DN.

## 4 Discussion

In this comprehensive study, leveraging extensive GWAS datasets, we employed the bidirectional two-sample MR method to scrutinize the causal links between GM, metabolites, and DN. Our findings unveiled compelling insights: we identified that heightened levels of *Lachnospiraceae, Parasutterella, and Eubacterium* are causally associated with an increased risk of DN. Conversely, augmented abundance of *Coprococcus2, Sutterella, Faecalibacterium, Prausnitzii, and Bacteroides-vulgatus* is linked to a decreased risk of DN. After FDR correction, we identified a positive correlation between the average number of double bonds in a fatty acid chain and the risk of DN. Moreover, thorough sensitivity analyses reinforced the robustness and consistency of these findings, confirming their reliability and significance in elucidating the complex interplay between gut microbiota, metabolites, and DN pathogenesis.

DN is intricately linked to the composition of gut microbiota, the intestinal environment, and the permeability of the intestinal barrier. Uremic toxins, primarily endogenous compounds produced through microbial metabolism in the intestine, play a key role in this process (27). In DN pathology, the microbial balance within the intestines of affected individuals is disrupted, leading to a compromised intestinal barrier. Metabolism-induced urea raises intestinal pH levels, worsening mucosal damage and triggering epithelial barrier dysfunction. As a result, these enterotoxins enter systemic circulation, negatively impacting renal function (28). Notably, individuals with DN often reduce carbohydrate intake to manage blood glucose levels, which inadvertently promotes protein catabolism and the production of nitrogen-containing compounds. This leads to the accumulation of nitrogenous toxic byproducts, creating a harmful feedback loop. Thus, the “intestinal-renal axis” emerges as a critical driver in the pathogenesis and progression of DN.

The concept of the “intestinal-renal axis” draws inspiration from the theory of “intestinal-renal syndrome” initially proposed by Ritz (29) at the international dialysis conference in 2011. This theory illuminates a profound connection between the pathology and physiology of the intestine and kidney. It elucidates that the intestine serves not only as a vital site for human nutrition digestion and absorption but also stands as the largest immune organ within the human body. The intricate interplay between the human immune system and GM, orchestrated through the maintenance of the intestinal barrier and immune regulation, underscores the pivotal role of the intestine in systemic health. Conversely, the GM plays a pivotal role in fostering the maturation of lymphoid tissue integral to the human intestinal mucosa (30).

The study by Feng (31) highlighted an elevated abundance of members from the *Lachnospiraceae family* in patients diagnosed with DN, with a positive correlation observed between serum C-reactive protein (CRP) levels and *Lachnospiraceae* abundance. Additionally, the involvement of *Parasutterella* in ω3-fatty acid metabolism underscores its significance within the inflammatory milieu of the intestinal mucosa (32). Another investigation revealed that patients with end-stage renal disease exhibited the most pronounced elevation in *Firmicutes bacterial* abundance compared to healthy controls (33). Of particular note is the implication of *Coprococcus* in the induction of metabolic syndrome, oxidative stress, and inflammatory reactions, with its richness correlating with the onset of metabolic syndrome. Studies suggest that *Coprococcus* may disrupt the level and composition of SCFAs in the body, thereby precipitating adverse effects in diabetic patients, including impaired islet cell function and reduced insulin sensitivity (33). Furthermore, the heightened presence of *Sutterella* in individuals with DN has been associated with a notable increase in circulating lipopolysaccharides (LPS), thereby fostering inflammatory cascades (34). These findings underscore the intricate interplay between GM composition and renal health, shedding light on potential mechanisms contributing to the pathogenesis of DN and highlighting avenues for therapeutic intervention.

On the contrary, *Faecalibacterium* emerges as a noteworthy butyrate-producing microorganism residing within the gastrointestinal tract (35). Zhang (36) elucidated its capacity to downregulate Toll-like receptor 3 (TLR3) and Toll-like receptor 4 (TLR4) expression, thereby inhibiting the nuclear factor-kB (NF-kB) pathway and eliciting an anti-inflammatory effect on colonic epithelial cells (37). This anti-inflammatory action underscores the potential therapeutic significance of *Faecalibacterium* in mitigating inflammatory processes within the gut. Furthermore, Bacteroides, a prevalent constituent of the GM, has garnered significant attention in the realm of obesity and aberrant lipid metabolism research. Studies have underscored the association between reduced *Bacteroides* abundance and an elevated *Firmicutes to Bacteroides ratio* with obesity and dyslipidemia (38). This highlights the intricate role of *Bacteroides* in modulating host metabolic processes and suggests its potential implications in the management of metabolic disorders.

In essence, the dysregulation of gut microbiota triggers a complex cascade of adverse effects, including compromised intestinal epithelial barrier integrity, increased inflammation, oxidative stress, reduced insulin sensitivity, renal fibrosis, and the progressive development of DN. These detrimental outcomes are primarily driven by the synthesis and release of key microbial metabolites, such as SCFAs, lipopolysaccharides (LPS), and enterogenous urotoxins. A wide range of intricate interactions between the human body and gut microbiota are mediated by microbial metabolites or small molecular byproducts produced through GM metabolism. Through the enzymatic breakdown of carbohydrates, proteins, and peptides by enzymes like urease, uric acid oxidase, and indole, GM produces a variety of metabolites, including SCFAs, hydrogen sulfide and its derivatives, bile acids, trimethylamine/trimethylamine oxide, and indane. Notably, metabolites such as indole/indophenol sulfate, endotoxin, and choline (39) intricately modulate the functionality of the intestinal epithelial barrier by regulating receptor expression and/or activating transcription factors, thereby influencing the initiation and progression of DN. Consequently, rectifying GM dysbiosis emerges as a highly promising therapeutic avenue for intervention, offering a tantalizing prospect for ameliorating the pathogenesis of DN and its associated complications.

The clinical implications of this investigation highlight the potential of oral probiotics as an effective intervention strategy for managing patients with DN. Several strengths enhance the validity and significance of this study. Firstly, the robust sample size serves as a strong safeguard against the confounding influence of extraneous variables, thereby increasing the credibility and reliability of the results. Secondly, the adoption of MR methodology effectively addresses the challenges of reverse causality and confounding biases common in observational studies. This approach not only strengthens the validity of the findings but also optimizes resource allocation, maximizing research efficiency. Thirdly, this study represents a pioneering effort to unravel the genetic mechanisms governing the interaction between gut microbiota and metabolites in the context of DN. Fourthly, the careful use of various effect models enables a thorough exploration of sensitivity, pleiotropy, and heterogeneity, resulting in more robust and nuanced outcomes. This multifaceted approach ensures a comprehensive understanding of the complex dynamics at play, further enriching the scientific discussion surrounding DN management strategies.

Nonetheless, several limitations must be considered. First, as the data used in this study is derived from a European population, caution should be exercised regarding the generalizability of the findings to non-European populations due to potential population stratification issues. Future research should aim to include larger, more diverse GWAS populations to validate and broaden the scope of these conclusions. Second, despite efforts to control for confounding factors related to DN, it is important to note a limitation concerning the MR assumption of genotype independence from GM-metabolite-DN confounders. This could introduce unmeasured confounding, warranting careful interpretation of the results. Third, the lack of detailed data, such as age and gender, limits our ability to conduct subgroup analyses, thus constraining the depth of our insights. Incorporating such demographic details in future studies would enhance the robustness and applicability of our findings. Fourth, despite thorough efforts to identify and address abnormal variations, the potential influence of unobserved pleiotropy on our results cannot be entirely ruled out. Further exploration and sensitivity analyses are needed to address this issue and further strengthen the reliability of our findings (40). Fifthly, due to limitations imposed by available public databases, our investigation was restricted to verifying the reverse causality between GM and DN, without exploring the reverse causality of metabolites. Future research efforts should aim to elucidate these intricate relationships and provide additional theoretical support for understanding the mechanisms underlying the “intestine-kidney” axis. Lastly, future research endeavors stand to benefit from the integration of comprehensive covariate data through sequencing verification, which would offer a more holistic understanding of the complex interplay among GM, metabolites, and DN.

## 5 Conclusion

Indeed, our study underscores a causal association between GM, metabolites, and DN, highlighting *Coprococcus2, Sutterella, Faecalibacterium, Prausnitzii, and Bacteroides-vulgatus* as protective factors against DN. Increasing the abundance of these specific GM species shows promise for reducing the incidence and improving the clinical management of DN, potentially paving the way for probiotic-based interventions in DN treatment strategies. Furthermore, this approach could strengthen the theoretical foundation of the “intestine-kidney” axis.

## Data Availability

The original contributions presented in the study are included in the article/**Supplementary Material**. Further inquiries can be directed to the corresponding author/s.

## Supplementary material

Supplementary Figure 1 Funnel plots of sensitivity analysis.

Supplementary Figure 2 Forest plots of LOO sensitivity analysis. The black dot signifies the DN with increased standard deviation (SD) in GM or metabolites, generated by using each SNP as a separate tool variable. The red dot represents the causal estimation of all SNP combinations by different MR methods. The horizontal line segment represents the 95% CI. It illustrates the Inverse Variance Weighted (IVW) causal estimate and how the overall estimate (red horizontal line) was disproportionately driven, influenced by the removal of a single variant (black horizontal line).

Supplementary Table 1 Specific information of IVs used in MR analysis.

Supplementary Table 2 Results of sensitivity analysis.
