# Supplementary material for: Elucidating the causal relationship between gut microbiota, metabolites, and diabetic nephropathy in European patients: Revelations from genome-wide bidirectional mendelian randomization analysis
Source: Front Endocrinol (Lausanne). 2025 Jan 8;15:1391891. doi: 10.3389/fendo.2024.1391891 (PMC11750691; doi:10.3389/fendo.2024.1391891)

Supplementary Figure 1

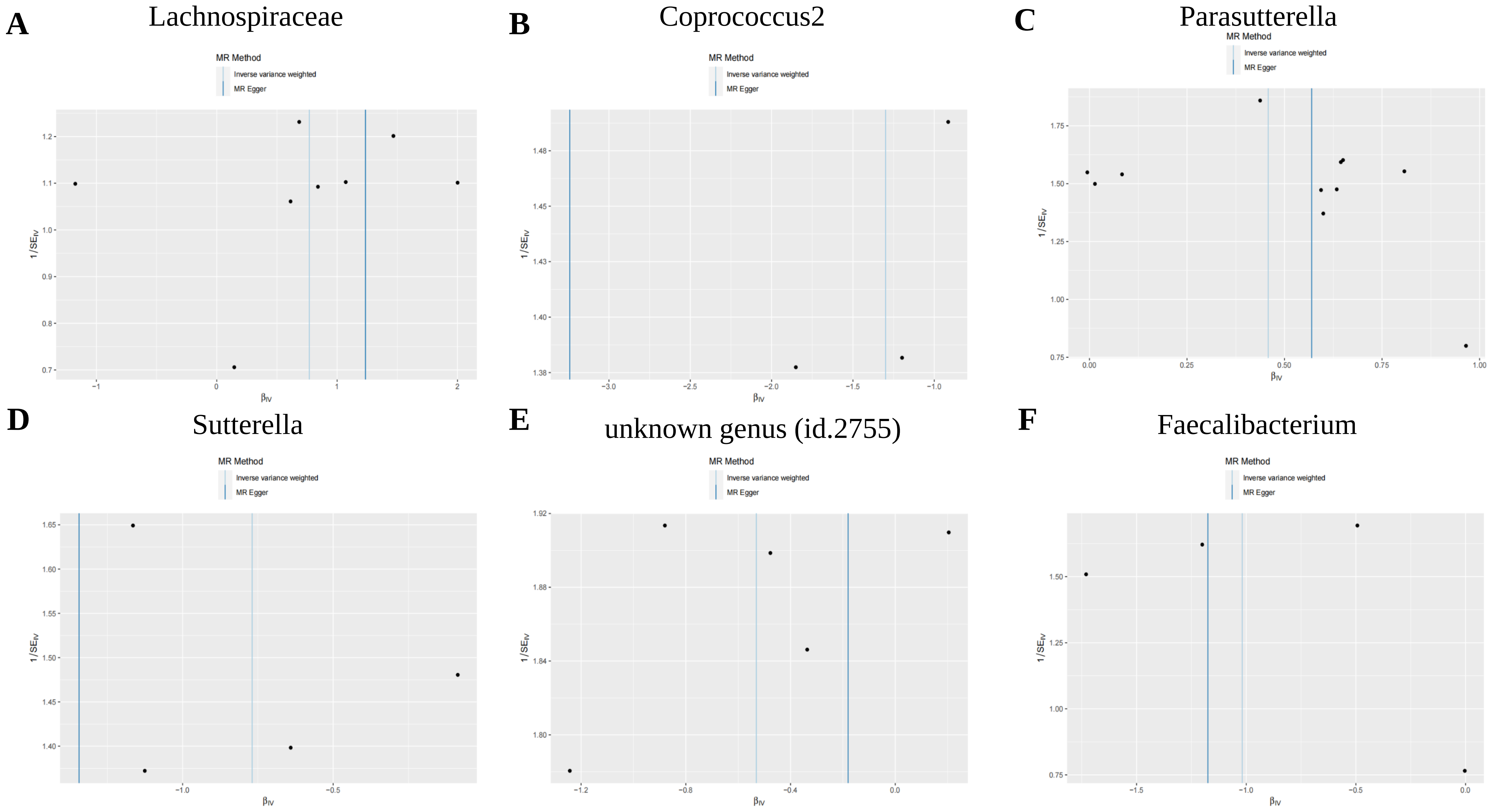

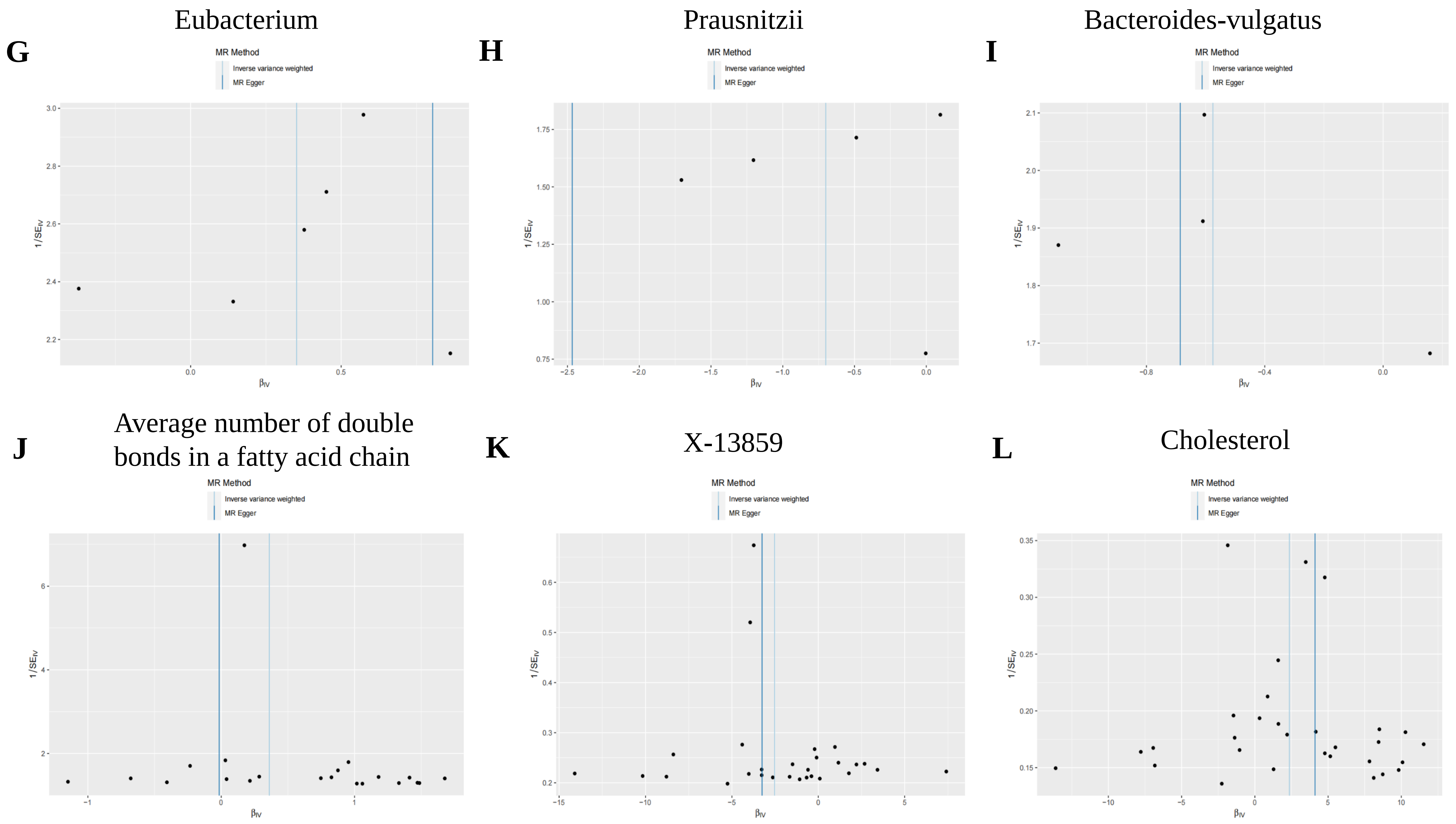
G
Eubacterium
H
Prausnitzii
I
Bacteroides-vulgatus
J
Average number of double bonds in a fatty acid chain
K
X-13859
L
Cholesterol
MR Method
Inverse variance weighted
MR Egger
1/SE$_{IV}$
β$_{IV}$

M Methionine

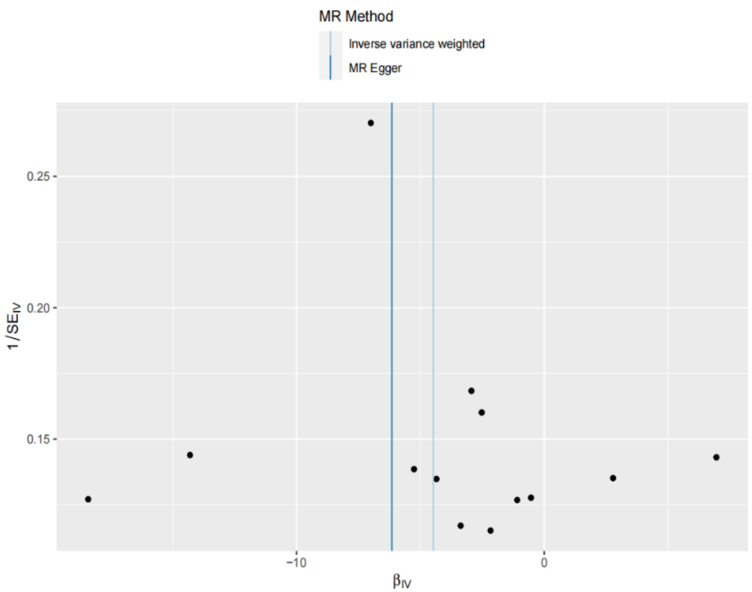

N Glycodeoxycholate

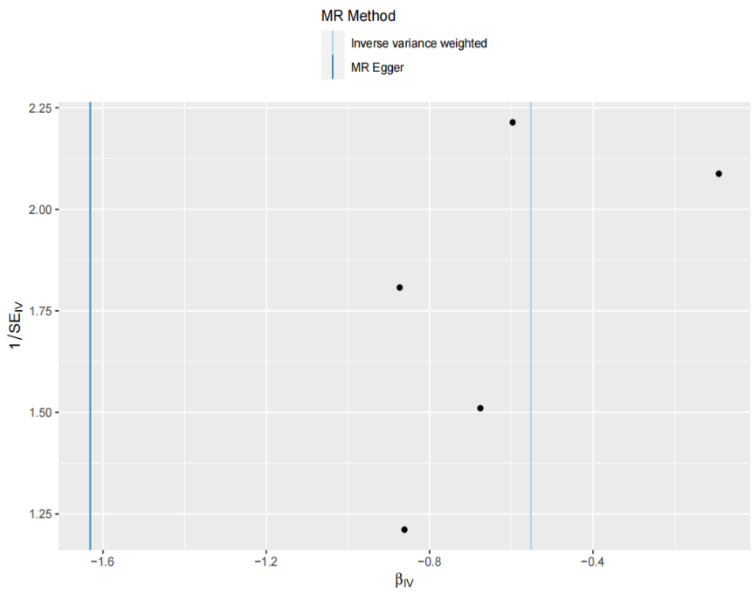

O X-06351

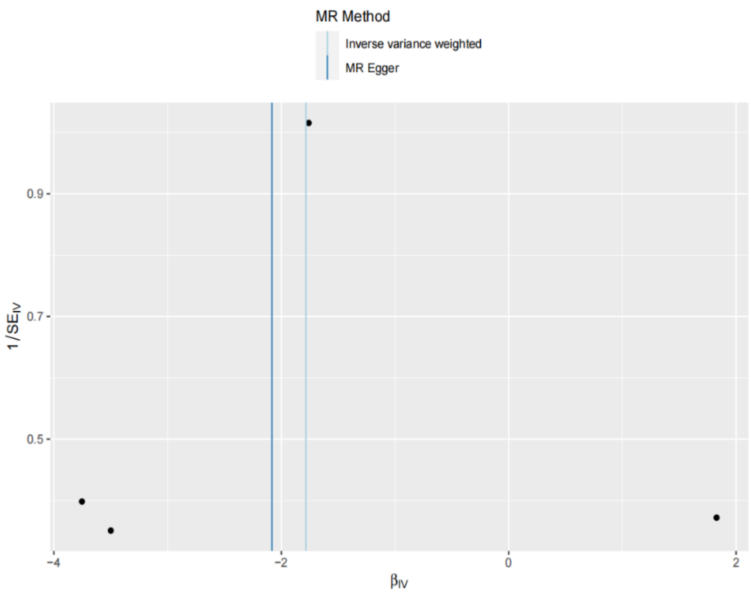

P 1-stearoylglycerol (1-monostearin)

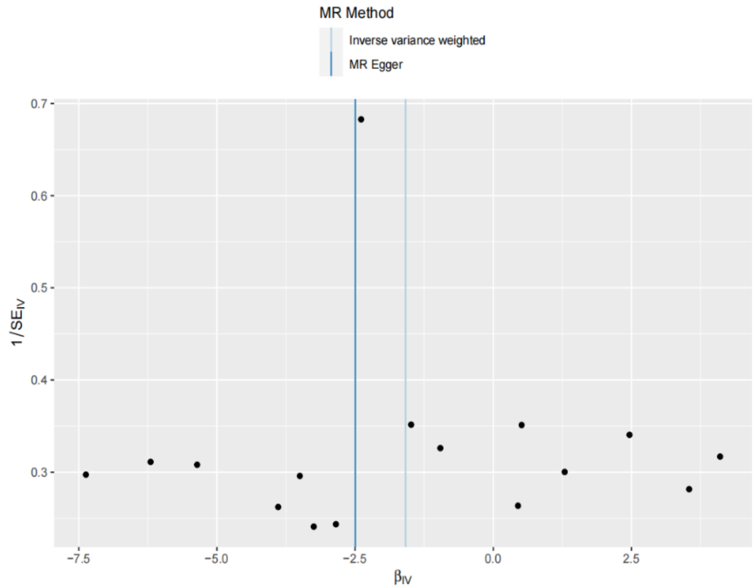

Q Pyridoxate

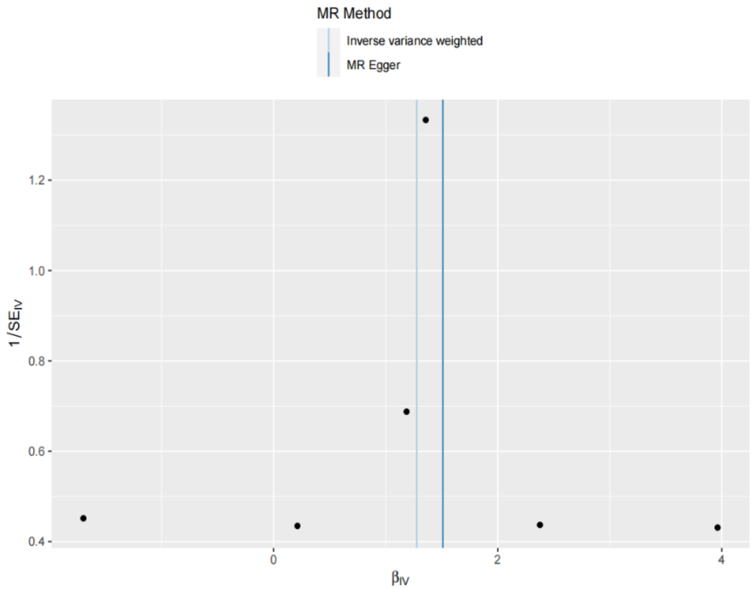

R Hexanoylcarnitine

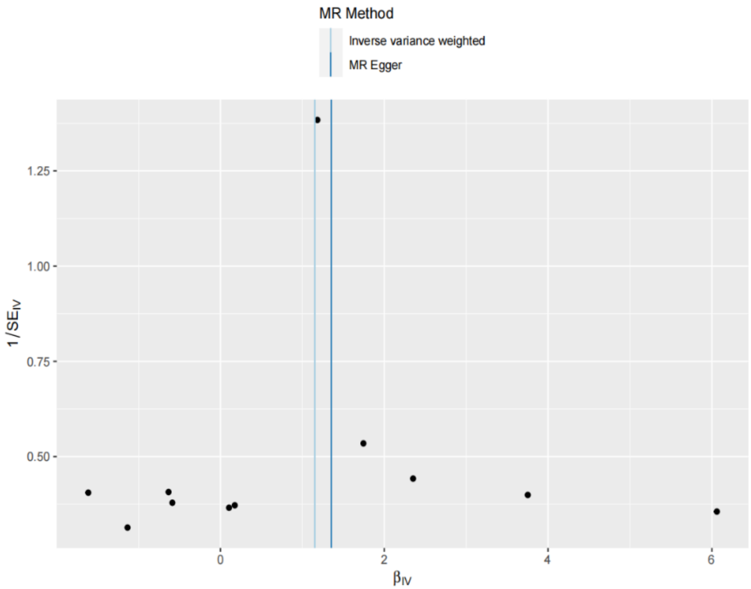

S X-12007

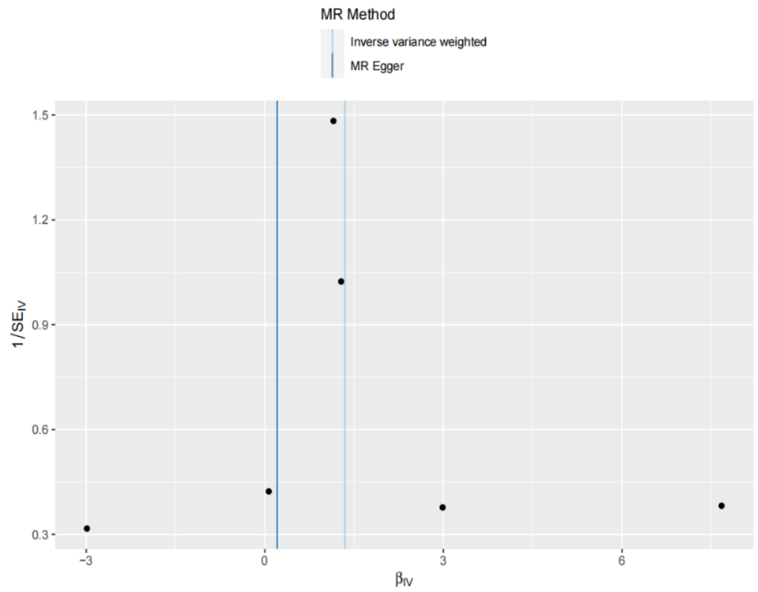

T Octanoylcarnitine

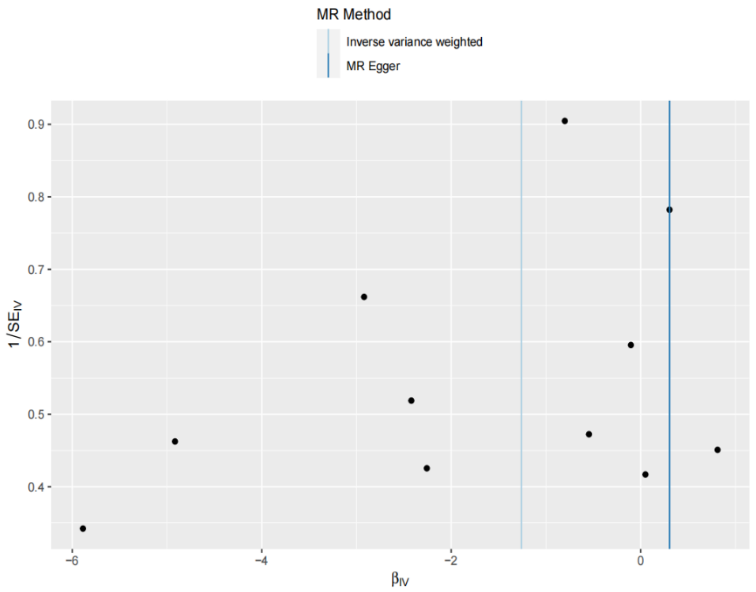

U X-12734

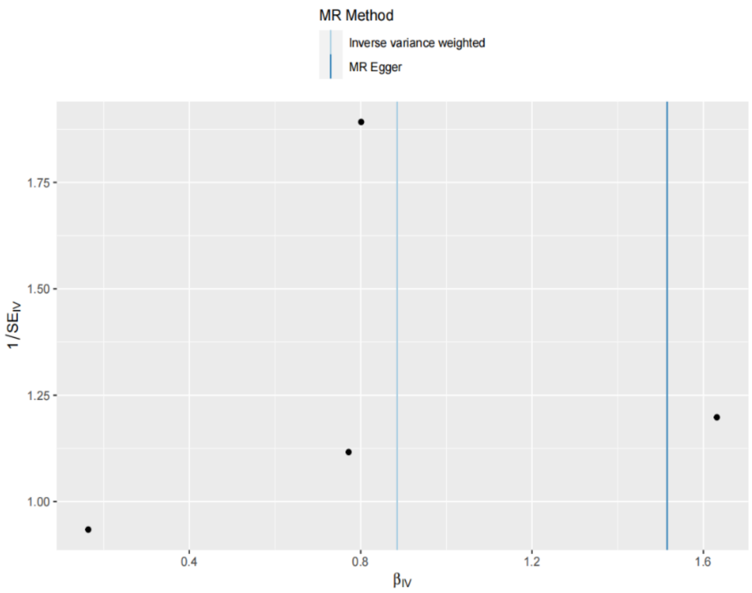

V Glycoproteins

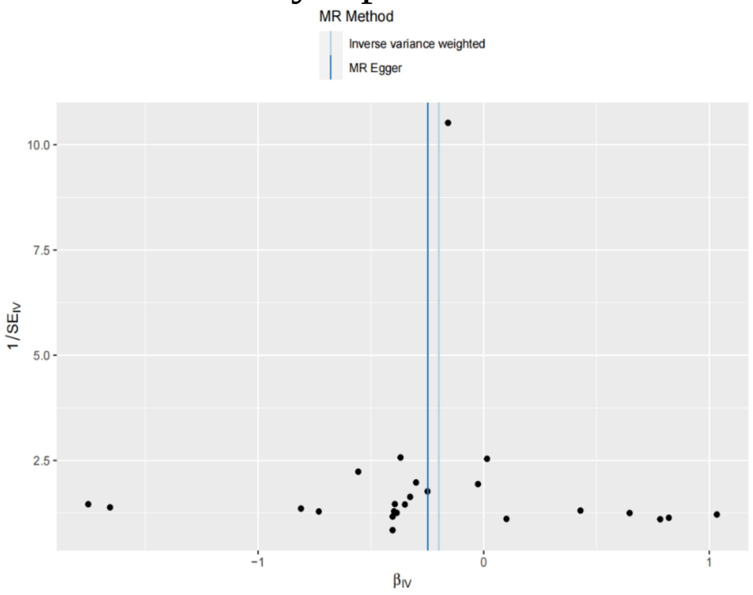

W Concentration of small HDL particles

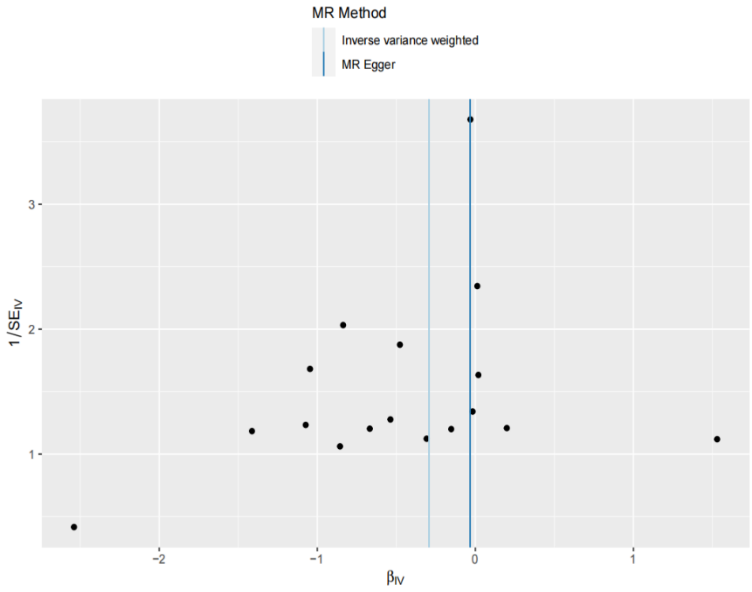

Supplement: Supplementary Figure 1 — Funnel plots of sensitivity analysis. [file DataSheet1.pdf]
