# Supplementary material for: Elucidating the causal relationship between gut microbiota, metabolites, and diabetic nephropathy in European patients: Revelations from genome-wide bidirectional mendelian randomization analysis
Source: Front Endocrinol (Lausanne). 2025 Jan 8;15:1391891. doi: 10.3389/fendo.2024.1391891 (PMC11750691; doi:10.3389/fendo.2024.1391891)

Supplementary Figure 2

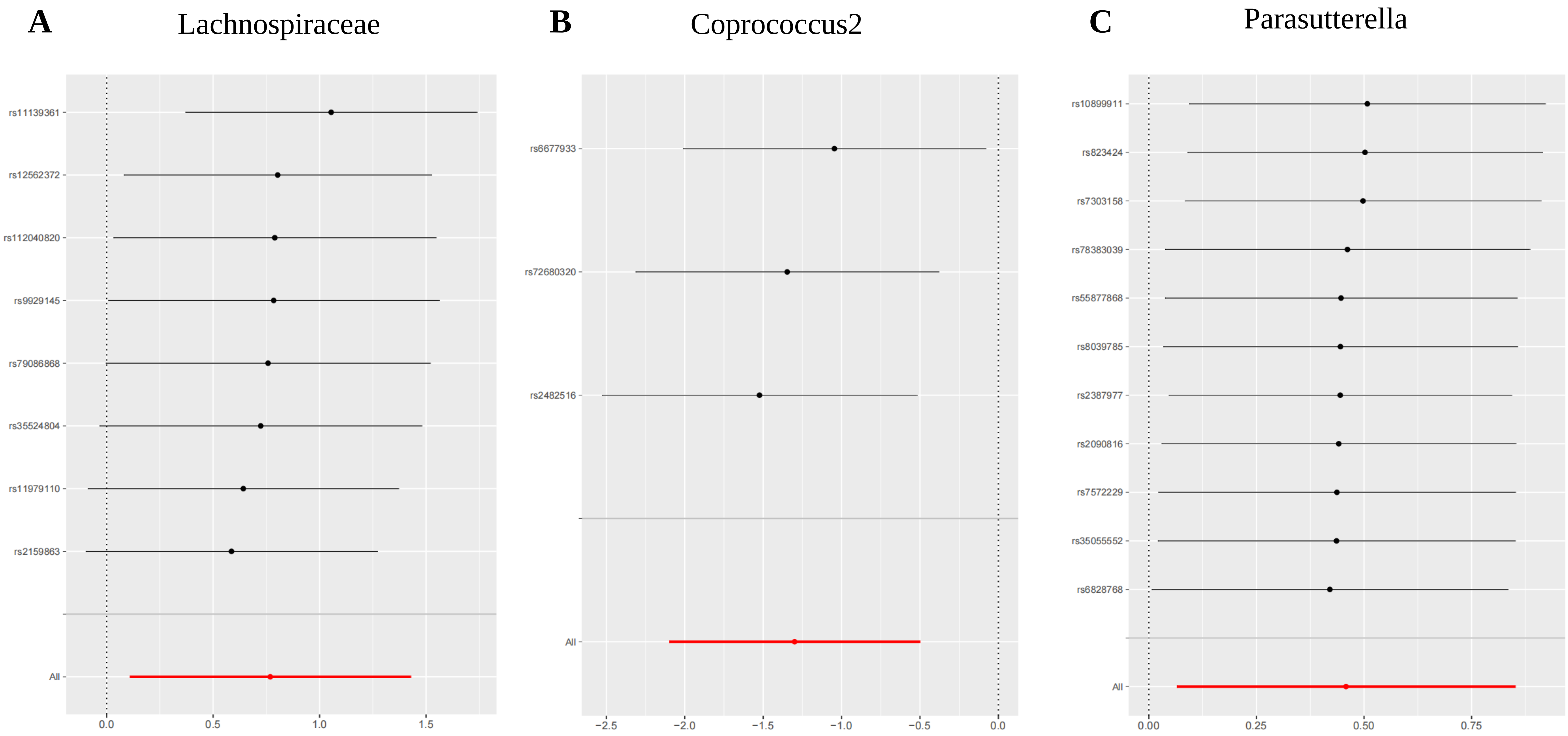

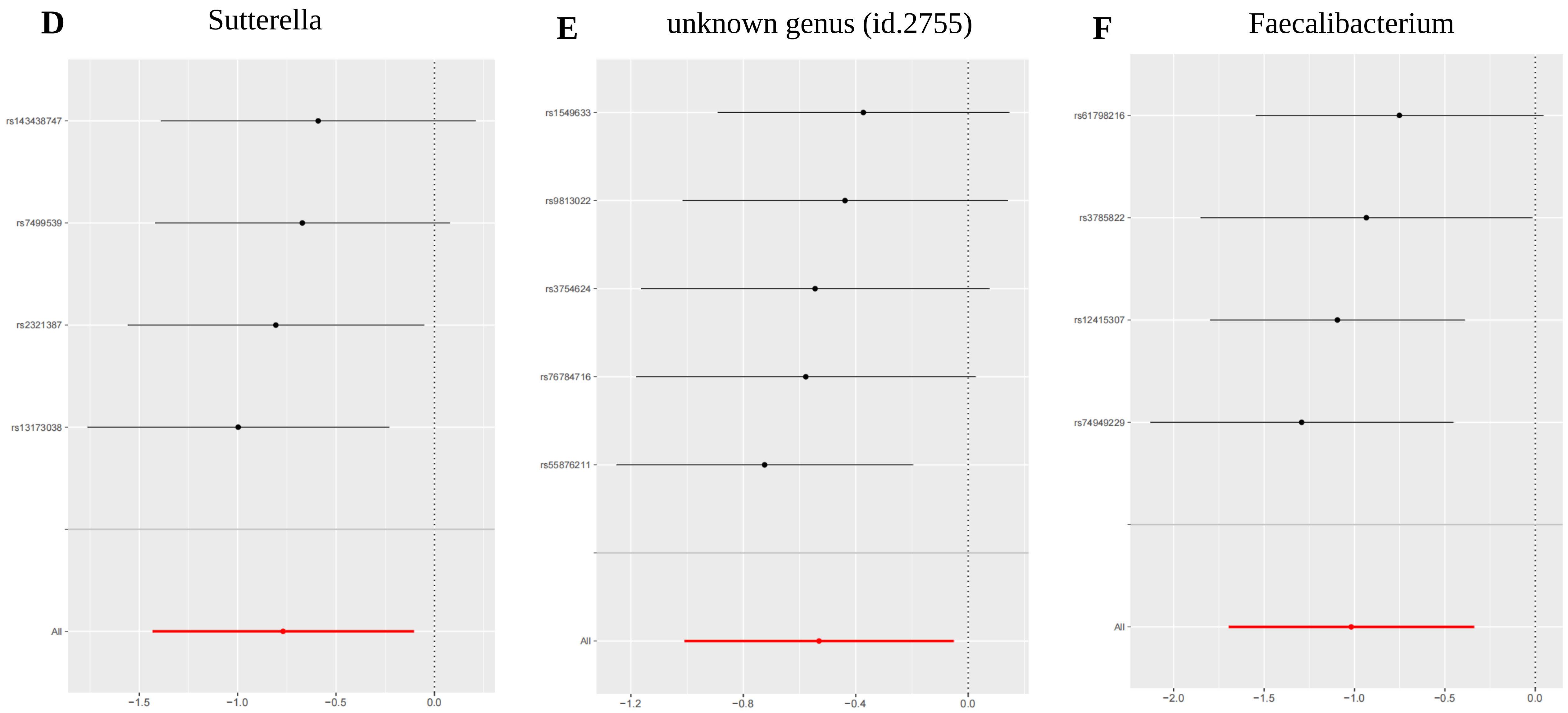
D
Sutterella
rs143438747
rs7499539
rs2321387
rs13173038
All
−1.5
−1.0
−0.5
0.0
E
unknown genus (id.2755)
rs1549633
rs9813022
rs3754624
rs76784716
rs55876211
All
−1.2
−0.8
−0.4
0.0
F
Faecalibacterium
rs61798216
rs3785822
rs12415307
rs74949229
All
−2.0
−1.5
−1.0
−0.5
0.0

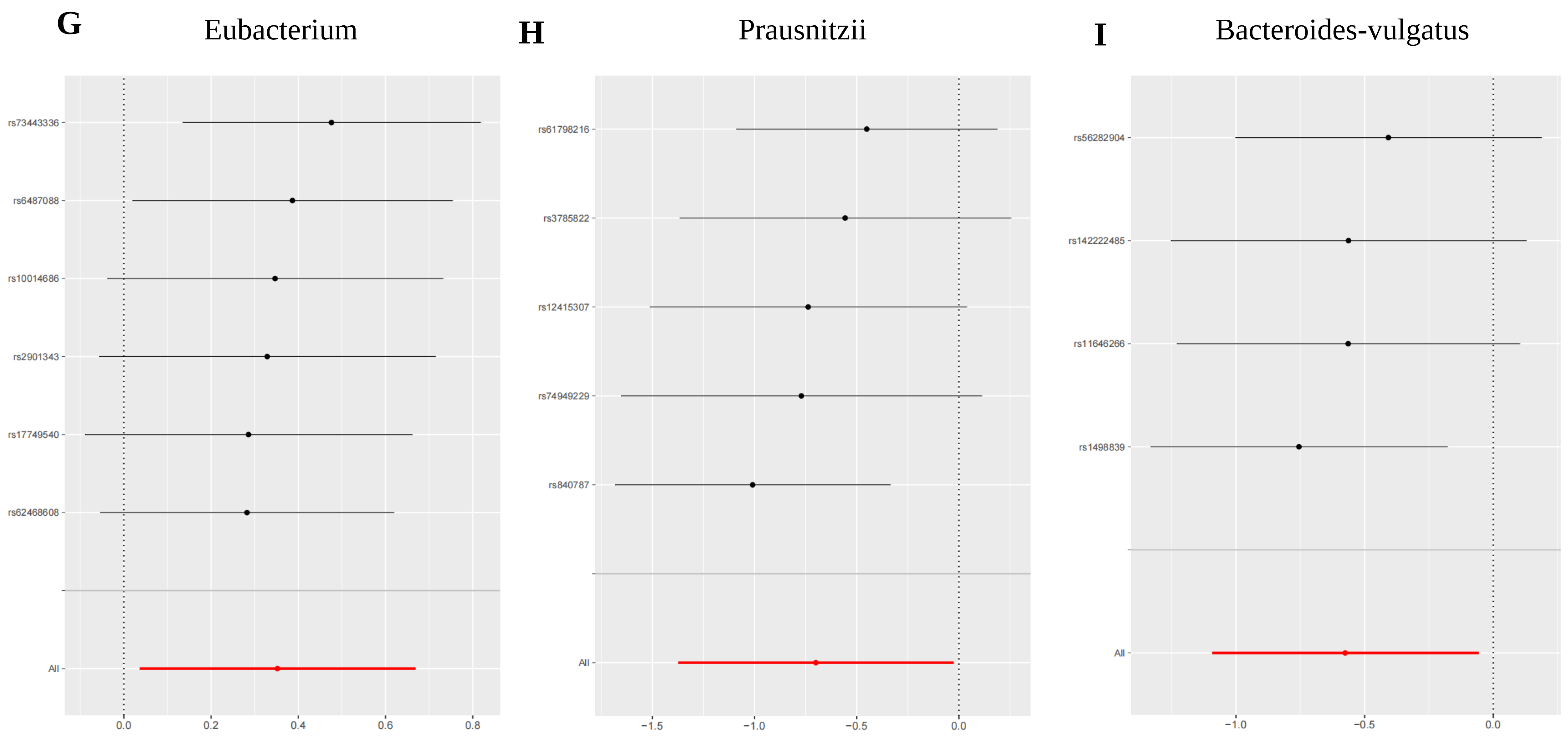
G
Eubacterium
rs73443336
rs6487088
rs10014686
rs2901343
rs17749540
rs62468608
All
0.0
0.2
0.4
0.6
0.8
H
Prausnitzii
rs61798216
rs3785822
rs12415307
rs74949229
rs840787
All
−1.5
−1.0
−0.5
0.0
I
Bacteroides-vulgatus
rs56282904
rs142222485
rs11646266
rs1498839
All
−1.0
−0.5
0.0

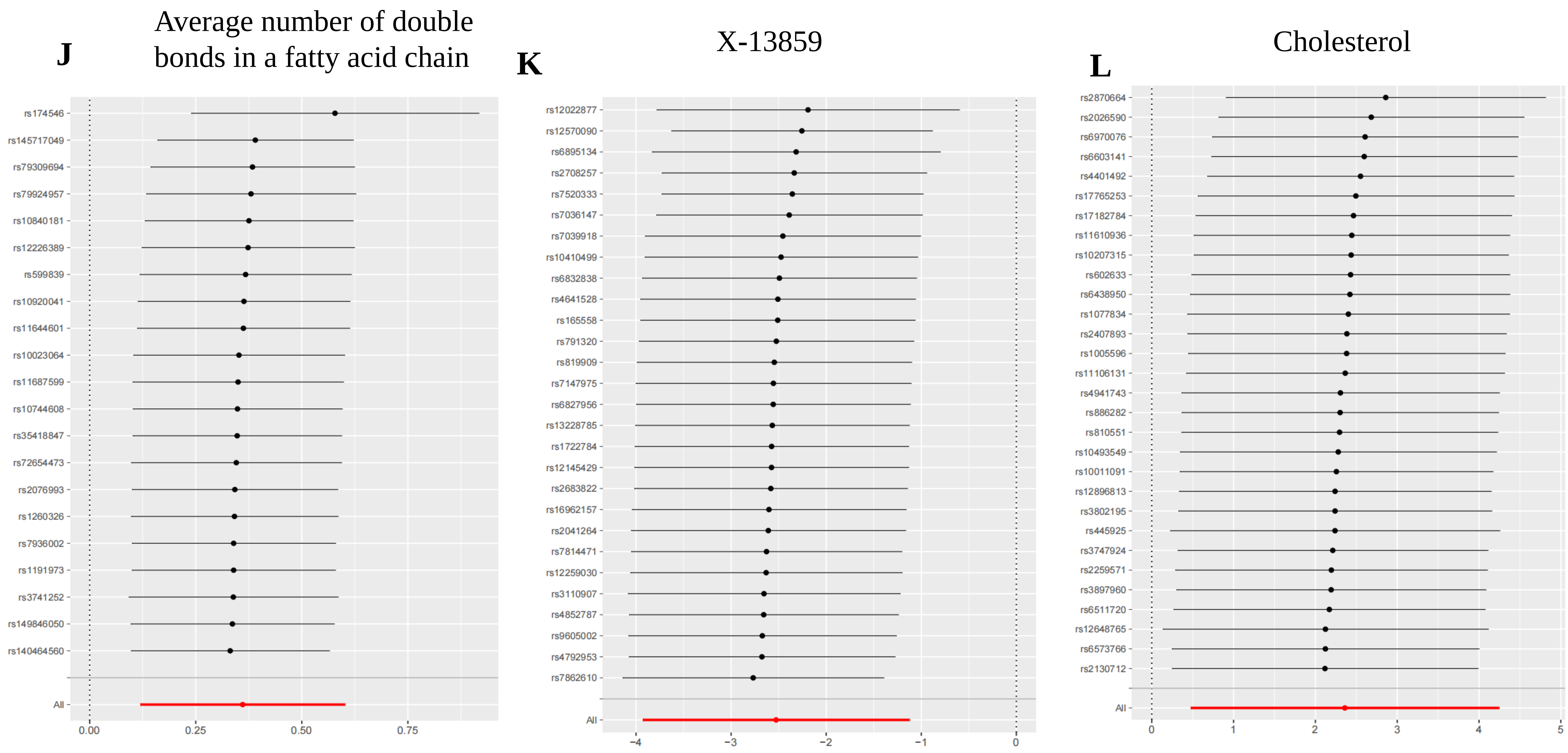
J
Average number of double bonds in a fatty acid chain
rs174546
rs145717049
rs79309694
rs79924957
rs10840181
rs12226389
rs599839
rs10920041
rs11644601
rs10023064
rs11687599
rs10744608
rs35418847
rs72654473
rs2076993
rs1260326
rs7936002
rs1191973
rs3741252
rs149846050
rs140464560
All
0.00
0.25
0.50
0.75
K
X-13859
rs12022877
rs12570090
rs6895134
rs2708257
rs7520333
rs7036147
rs7039918
rs10410499
rs6832838
rs4641528
rs165558
rs791320
rs819909
rs7147975
rs6827956
rs13228785
rs1722784
rs12145429
rs2683822
rs16962157
rs2041264
rs7814471
rs12259030
rs3110907
rs4852787
rs9605002
rs4792953
rs7862610
All
−4
−3
−2
−1
0
L
Cholesterol
rs2870664
rs2026590
rs6970076
rs6603141
rs4401492
rs17765253
rs17182784
rs11610936
rs10207315
rs602633
rs6438950
rs1077834
rs2407893
rs1005596
rs11106131
rs4941743
rs886282
rs810551
rs10493549
rs10011091
rs12896813
rs3802195
rs445925
rs3747924
rs2259571
rs3897960
rs6511720
rs12648765
rs6573766
rs2130712
All
0
1
2
3
4
5

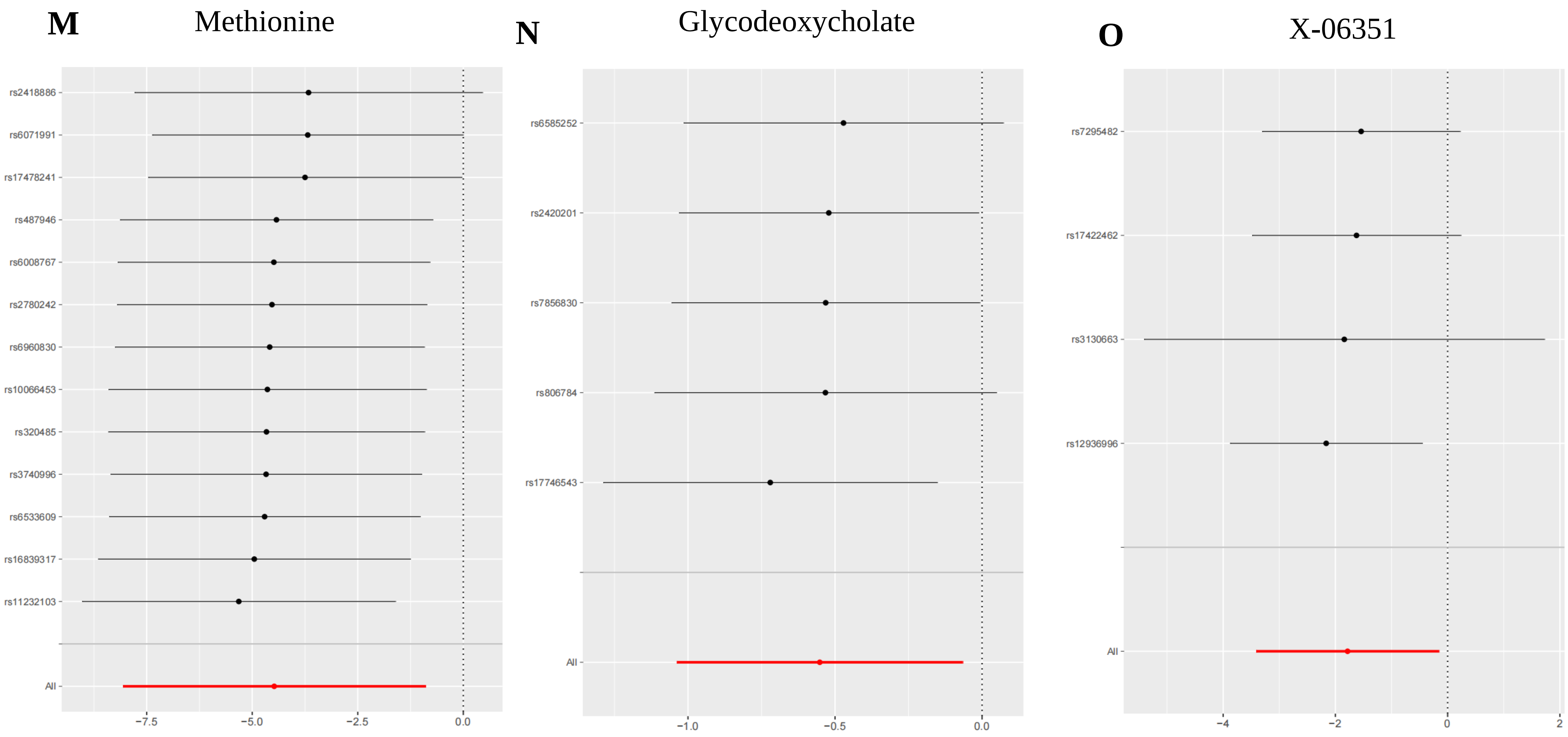
M
Methionine
rs2418886
rs6071991
rs17478241
rs487946
rs6008767
rs2780242
rs6960830
rs10066453
rs320485
rs3740996
rs6533609
rs16839317
rs11232103
All
−7.5
−5.0
−2.5
0.0
N
Glycodeoxycholate
rs6585252
rs2420201
rs7856830
rs806784
rs17746543
All
−1.0
−0.5
0.0
O
X-06351
rs7295482
rs17422462
rs3130663
rs12936996
All
−4
−2
0
2

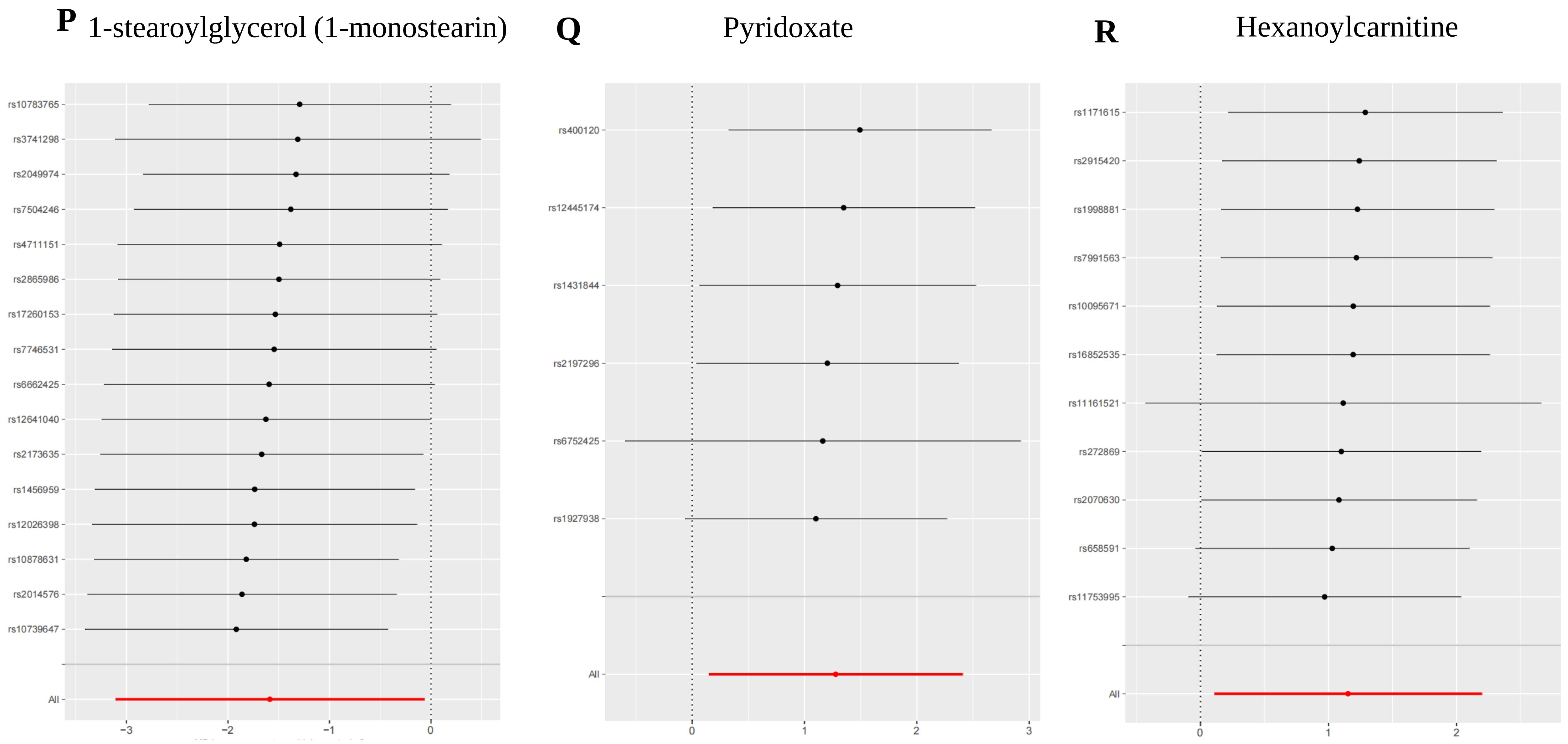
P
1-stearoylglycerol (1-monostearin)
rs10783765
rs3741298
rs2049974
rs7504246
rs4711151
rs2865986
rs17260153
rs7746531
rs6662425
rs12641040
rs2173635
rs1456959
rs12026398
rs10878631
rs2014576
rs10739647
All
−3
−2
−1
0
Q
Pyridoxate
rs400120
rs12445174
rs1431844
rs2197296
rs6752425
rs1927938
All
0
1
2
3
R
Hexanoylcarnitine
rs1171615
rs2915420
rs1998881
rs7991563
rs10095671
rs16852535
rs11161521
rs272869
rs2070630
rs658591
rs11753995
All
0
1
2

S X-12007

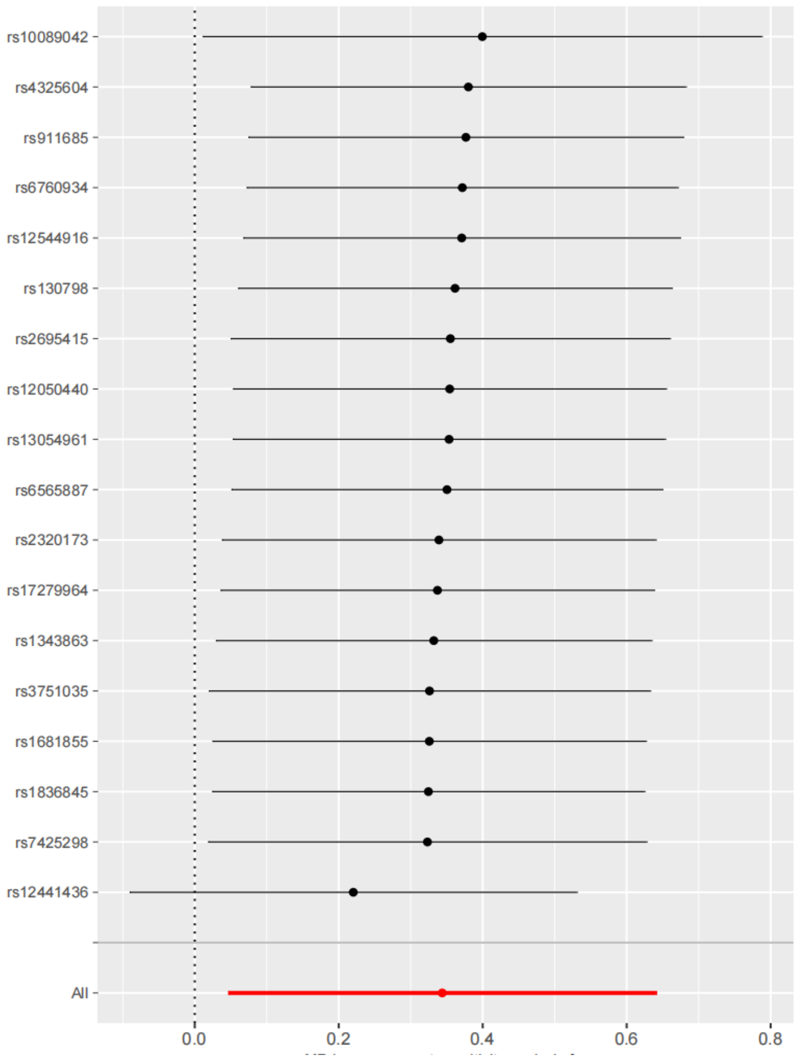

T Octanoylcarnitine

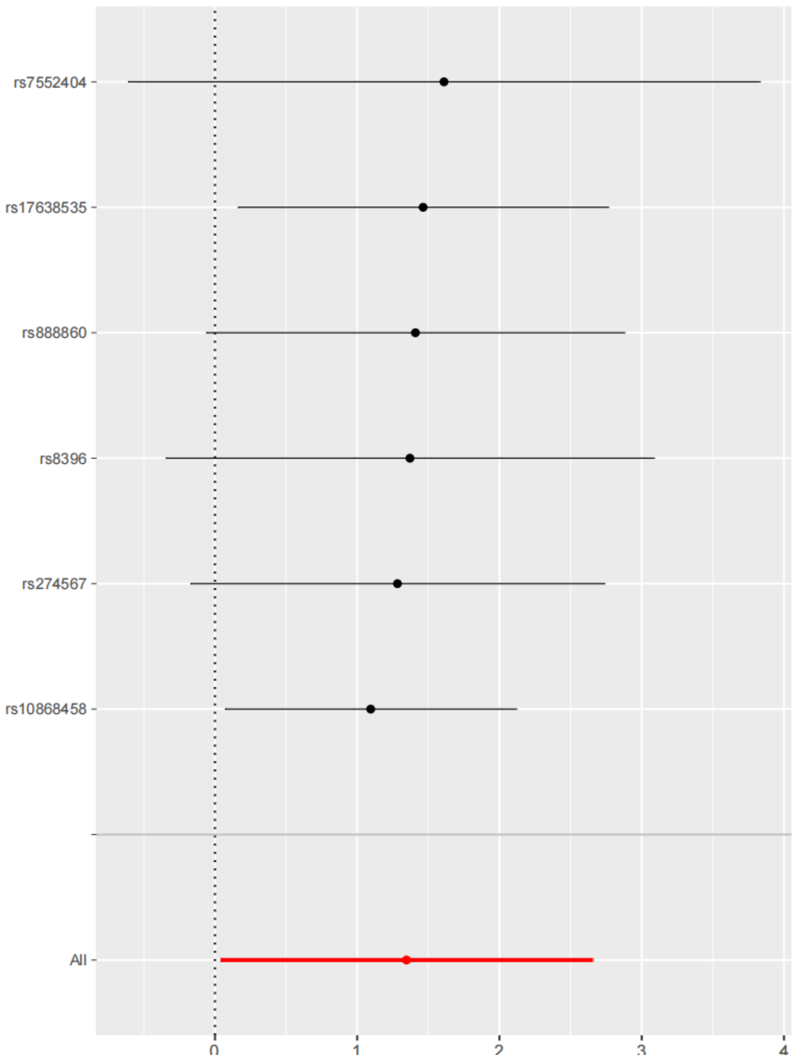

U X-12734

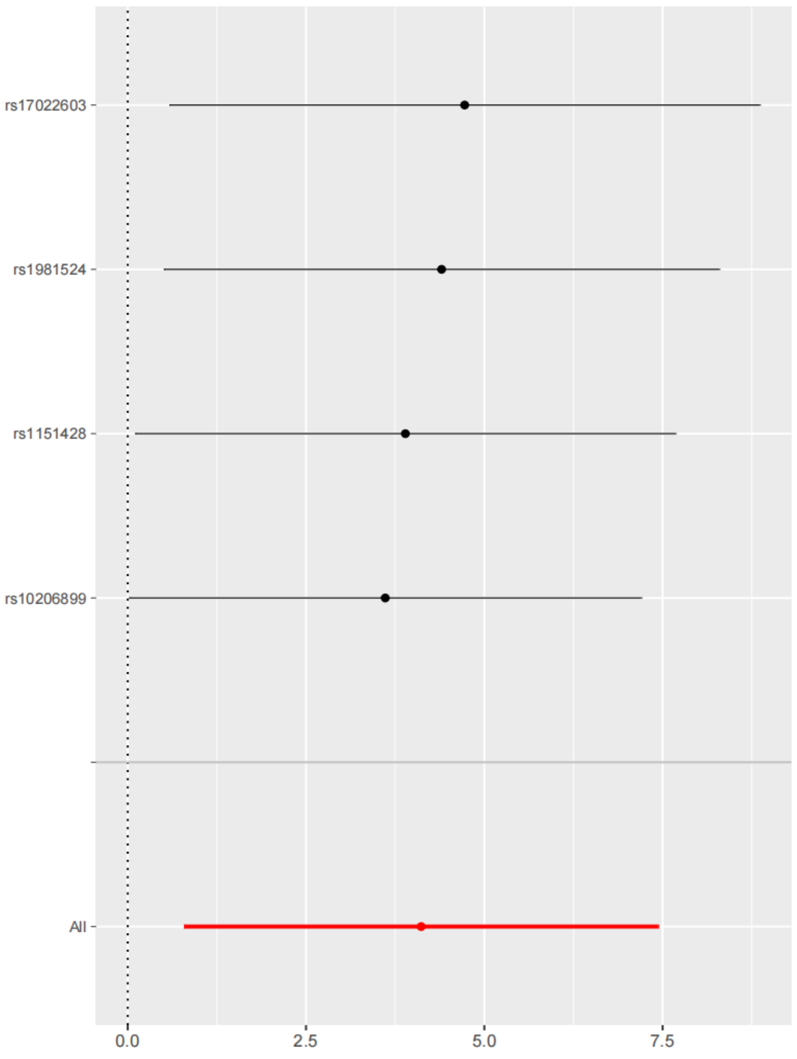

V Glycoproteins

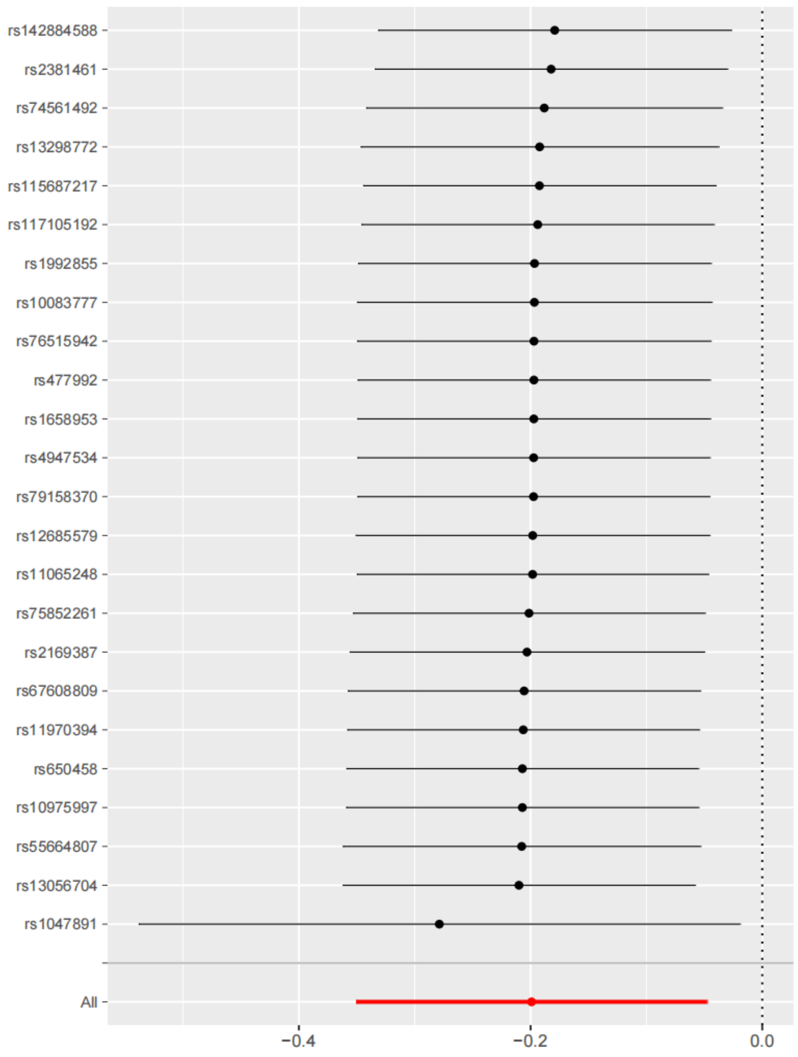

W Concentration of small HDL particles

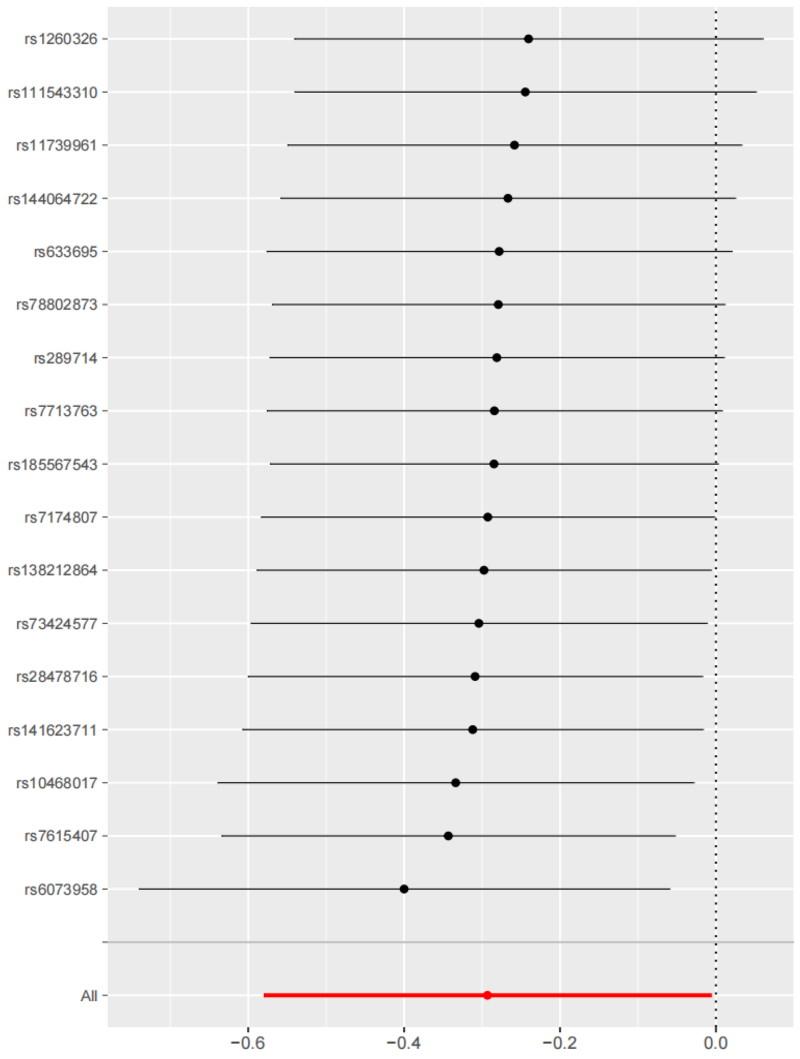

Supplement: Supplementary Figure 2 — Forest plots of LOO sensitivity analysis. The black dot signifies the DN with increased standard deviation (SD) in GM or metabolites, generated by using each SNP as a separate tool variable. The red dot represents the causal estimation of all SNP combinations by different MR methods. The horizontal line segment represents the 95% CI. It illustrates the Inverse Variance Weighted (IVW) causal estimate and how the overall estimate (red horizontal line) was disproportionately driven, influenced by the removal of a single variant (black horizontal line). [file DataSheet2.pdf]
